# Novel Impact of Colchicine on Interleukin-10 Expression in Acute Myocardial Infarction: An Integrative Approach

**DOI:** 10.3390/jcm13164619

**Published:** 2024-08-07

**Authors:** Saskia Dyah Handari, Mohammad Saifur Rohman, Djanggan Sargowo, Aulanni’am, Ricardo Adrian Nugraha, Bayu Lestari, Delvac Oceandy

**Affiliations:** 1 Doctoral Program of Medical Science, Faculty of Medicine, Brawijaya University, Malang 65145, Indonesia; 2 Medical Faculty, Ciputra University Surabaya, Surabaya 60271, Indonesia; 3 Department of Cardiology and Vascular Medicine, Faculty of Medicine, Brawijaya University—Dr. Saiful Anwar General Hospital, Malang 65145, Indonesia; ippoenk@ub.ac.id (M.S.R.); djanggan@yahoo.com (D.S.); 4 Department of Chemistry, Faculty of Sciences, Brawijaya University, Malang 65145, Indonesia; 5 Department of Cardiology and Vascular Medicine, Faculty of Medicine, Universitas Airlangga—Dr. Soetomo General Hospital, Surabaya 60115, Indonesia; ricardo.adrian.nugraha-2019@fk.unair.ac.id; 6 Division of Cardiovascular Sciences, University of Manchester, Manchester M13 9PL, UK; bayu.lestari@manchester.ac.uk (B.L.); delvac.oceandy@manchester.ac.uk (D.O.)

**Keywords:** colchicine, IL-10, inflammation, acute myocardial infarction, network pharmacology

## Abstract

**Background:** Inflammation plays a critical role in myocardial infarction as a critical process in the development of heart failure, involving the development of cardiac fibrosis. Colchicine is a well-established anti-inflammatory drug, but its scientific application in controlling post-acute myocardial infarction (AMI) inflammatory processes has not been established. IL-10 is a key cytokine in modulating inflammatory responses, underscoring its potential as a crucial therapeutic target of colchicine. The objective was to explore the protective role of IL-10 modulated by colchicine in myocardial healing and repair following AMI, particularly cardiac fibrosis. **Methods:** The predicted protein of colchicine was assessed using WAY2DRUG PASS as probability active value. Proteins associated with colchicine, cardiac fibrosis, and acute myocardial infarction were analyzed with DisGeNET and Open Target databases. Analysis and visualization of protein–protein interactions were conducted using STRING and Cytoscape. A 3T3 cell line treated with CoCl_2_ was used to mimic hypoxic. HIF-1α and IL-10 expression were measured by flow cytometry and analyzed using a one-way ANOVA test. This observational clinical trial examined acute myocardial infarction patients undergoing immediate and delayed primary percutaneous coronary interventions. Subjects were randomized into control groups receiving placebo and intervention groups treated with colchicine. Assessments occurred at 24 h and five days after the intervention. IL-10 expression in the clinical trial was measured by ELISA and analyzed using a *T*-test. **Results:** Colchicine demonstrates promising bioactivity in treating acute myocardial infarction, with notably activity values highlighting its probable role as a tubulin antagonist (0.744), beta-tubulin antagonist (0.673), and NOS2 inhibitor (0.529). Its primary action targets IL-10, with the protein–protein interactions analysis indicating interactions between IL-10 and key inflammatory mediators—IL-1β, IFN-γ, CCL2, TNF, and TGF-β1—during acute myocardial infarction and cardiac fibrosis. Hypoxic conditions in the CoCl_2_-induced 3T3 cell model show significantly elevated HIF-1α compared to controls (*p* < 0.0001). Colchicine use significantly increased IL-10 expression in CoCl_2_-treated cells (*p* < 0.0001) and in AMI patients within five days (*p* < 0.05). **Conclusions:** Colchicine may bolster the anti-inflammatory response post-myocardial infarction by activating IL-10 pathways in fibroblasts and in clinical settings, potentially reducing inflammation after AMI. Further investigation into broader aspects of this pathway, particularly in cardiac fibroblasts, is required.

## 1. Introduction

Cardiovascular disease, including coronary heart disease and stroke, is a prevalent non-communicable disease worldwide, responsible for approximately 17.8 million deaths in 2017, with more than three-quarters occurring in low-to-middle-income countries [1]. The incidence of heart and vascular diseases in Indonesia has been steadily rising over the years, making it the leading cause of death in the country. It is estimated that around 15 out of 1000 people in Indonesia suffer from heart disease [2]. The prevalence of acute myocardial infarction (AMI) approaches three million people worldwide each year [3].

Acute coronary syndrome resulting from coronary artery occlusion leads to ischemic or hypoxic conditions in the heart [4]. Heart disease is characterized by the enlargement of the myocardial infarction site during the acute phase, followed by left ventricular remodelling (VR), including dilation, fibrosis, and heart dysfunction [5]. Ventricular remodelling plays a significant role in the transition from myocardial infarction (MI) to heart failure (HF) mediated by inflammation [6]. The healing and prevention of detrimental VR tissue involve fibroblast proliferation, migration, and phenotypic transformation. Thus, modulating myocardial fibrosis levels after AMI is crucial to delaying or improving VR [7]. Polymorphonuclear cells are the first inflammatory cells recruited to the area of infarction and occurs within a few hours after MI. Polymorphonuclear cells are essentially regulatory early left ventricular remodelling response [8].

Inflammation control to enhance heart function following an acute myocardial infarction injury can be observed through several biomarkers, such as galectin-3, type III pro-collagen peptide (PIIINP), and IL-10 [9]. Cytokine therapy for reducing inflammation has been widely and successfully used in conditions such as rheumatoid arthritis and is currently an area of investigation for MI and HF treatment. Cardiovascular remodelling is a fluctuating process with observable clinical outcomes, making cytokines a potential therapeutic target [6]. IL-10 can improve left ventricular function, reduce infarction size, and mitigate infarct wall thinning [5].

Primary percutaneous coronary intervention (PCI) is the preferred reperfusion therapy for AMI [10]. However, the proportion of PCI remains low in some countries and regions [11]. PCI technology may alleviate atherosclerotic vessel narrowing or thrombosis, but exercise tolerance may decrease after revascularization [8].

Colchicine is an alkaloid extracted from plants of the *Colchicum* genus and is known to inhibit the inflammatory process [12]. Colchicine has relatively minor side effects, is readily available and is cost-effective [13]. Colchicine can reduce the expression of TNF-α receptors in macrophages and lower cytokines such as IL-1β, IFNγ, IL-18, and IL-6. Some studies suggest that colchicine plays a role in reducing the risk of recurrent MI. Colchicine can lower high-sensitivity C-reactive protein (HsCRP) levels and mean platelet volume (MPV) in AMI [14]. Colchicine triggers apoptosis in liver cells to inhibit hepatic fibrosis [15].

We developed three sequences of study: in silico, in vitro, and clinical trials. Since in vitro and in silico studies are indeed seen as an important step prior to conducting clinical trial studies in our research. An in silico study with a computational approach to evaluate the pharmacological properties of colchicine in preventing excessive inflammation in heart disease, in line with experimental advances, employs bioinformatics tools that allow the meta-search of databases to analyze biological interaction signal networks. This approach saves time and resources for laboratory-scale research [16]. To date, no scientific study has revealed the role of colchicine in postacute myocardial infarction excessive inflammation via IL-10 levels. Therefore, this research aims to analyze the effects of colchicine on reducing excessive inflammation postmyocardial infarction through IL-10 using both in silico and in vitro studies.

## 2. Materials and Methods

### 2.1. In Silico Study

#### 2.1.1. Prediction of Colchicine Potential Using a Structure–Activity Relationship (SAR) Approach

The Simplified Molecular-Input Line-Entry System (SMILES) profile and the structure of colchicine were obtained from the PubChem database (https://pubchem.ncbi.nlm.nih.gov/, accessed on 17 February 2024). Colchicine’s potential as an anti-inflammatory agent related to cardiac fibrosis and myocardial infarction was then analyzed using WAY2DRUG PASS prediction (http://www.pharmaexpert.ru/passonline/predict.php, accessed on 17 February 2024) by inserting its SMILES structure.

Structure–activity relationship (SAR) analysis was carried out using WAY2DRUG Pass Prediction to compare the input compound with known compounds that exhibit specific potential. The more similar the structures of the compounds are, the higher the prediction value. Compounds with high similarity typically share potential, as depicted by the probability to be active (Pa) value. A Pa value exceeding 0.7 indicates that the compound is predicted to have high potential as an anti-inflammatory agent due to its resemblance to compounds in the database that are known to be effective therapies. If 0.5 < Pa < 0.7, it suggests a lower likelihood of finding activity in further research, but the compound is not closely related to known pharmaceuticals. When Pa < 0.5, the chances of discovering activity in experiments become even slimmer, but if confirmed, the compound may represent a new chemical entity. Higher Pa values indicate better accuracy. This study used a reference range of 0.5 < Pa < 0.7 [17].

#### 2.1.2. Prediction of Target and Gene Disease Association with Cardiac Fibrosis (CF) and Acute Myocardial Infarction (AMI)

The target analysis of colchicine was performed using the Comparative Toxicogenomics Database (CTD) (http://ctdbase.org/, accessed on 17 February 2024) and DIGEP-Pred (http://www.way2drug.com/ge/, accessed on 17 February 2024) by entering colchicine’s SMILES structure. Genes and proteins associated with cardiac fibrosis and acute myocardial infarction were obtained from the DisGeNET and Open Target databases, accessed on 5 July 2023 [18]. Targets related to diseases and colchicine were then mapped using a Venn diagram to identify target intersections. Each identified target was annotated for its function using the Biological Function in the Database for Annotation, Visualization, and Integrated Discovery (DAVID) (https://david.ncifcrf.gov/, accessed on 29 May 2023 [19].

Based on Fisher’s exact test, the false discovery rate (FDR) was used as a statistical test method in the DAVID database. To validate the DAVID results, Network Analyst, commonly used for gene expression, functional analysis, and transcription factor analysis, was utilized [20].

#### 2.1.3. Pharmacology Network Analysis

A comprehensive list of target proteins, associated with cardiac fibrosis (CF) and acute myocardial infarction (AMI), was compiled from two distinct databases. This collection of proteins was then inputted into the ‘multiple protein’ feature of the STRING database (https://string-db.org/, accessed on 17 February 2024), a tool used for exploring protein–protein interactions and networks. A compilation of target proteins from both databases was input into the “multiple protein” feature of the STRING database (https://string-db.org/, accessed on 17 February 2024) [21]. The STRING database assesses protein–protein interactions [22]. Additional settings included choosing “*Homo sapiens*” as the organism, considering network edges as confidence, and setting the minimum needed interaction score to 0.9 for the physical subnetwork. After updating the network based on these additional settings, the visualization results were downloaded in the TSV (Tab-separated values) format in the “table/export” menu. The downloaded (.tsv) file was then imported into Cytoscape v.3.10.0 (accessed on 17 July 2023) for further network analysis using the “analyse network” procedure. The analysis network was used to identify the most functional and dominant proteins in the analyzed pathway, as depicted using parameters such as degree, betweenness centrality (BC), and closeness centrality (CC). These values help determine significant proteins. A higher BC value indicates a greater role of the protein in a pathway. The degree indicates interactions of the compound with other proteins (represented by nodes). A higher degree implies more interactions with other nodes. Closeness centrality calculates the role of a protein as a communicator or hub. The higher the closeness centrality value is, the greater the protein’s role in information and communication pathways. Degree helps identify the shortest pathways targetable in the path [23].

### 2.2. In Vitro Study

#### 2.2.1. Induction of Hypoxia in 3T3 Cell Line

To mimic the model of ischemia in vitro, we established the hypoxic fibroblast by treating the 3T3 cells with CoCl_2_. Cells were cultured in DMEM supplemented with 10% FBS and antibiotics at 37 °C in a humidified atmosphere with 5% CO_2_ and 95% air. To induce hypoxic conditions, fibroblasts were treated with CoCl_2_ (Sigma-Aldrich, St. Louis, MO, USA) at different concentrations of 300 μM for 24 h (dose optimization was performed) [24,25]. Colchicine was administered to 3T3 cell lines at a concentration of 10 µM. The dosage was based on the research protocol created by Wang et al. [26].

#### 2.2.2. Measurement of HIF-1α and IL-10 Expression

The expression of HIF-1α and IL-10 were measured by using the flowcytometry method. Briefly, the 3T3 cell line suspension was centrifuged at 2500 rpm for 5 min at a temperature of 10 °C. The supernatant was discarded, and the cell pellet was resuspended in 1 mL of PBS, divided into two microtubes (tubes A and B) of 300 µL each, and centrifuged again. The cell pellet was treated with 100 µL of BD hotfix/cytoperm fixative solution and incubated for 20 min. It was then mixed with 500 µL of BD Perm/Wash buffer for permeabilization and centrifuged at 2500 rpm at 10 °C for 5 min. After discarding the supernatant, the cell pellet was stained with FITC-conjugated anti-human HIF-1α or IL-10 antibodies and incubated for 20 min. The cells, now incubated with antibodies, were supplemented with 400 µL of PBS. The samples were analyzed using a FACS Calibur^TM^ flow cytometer [27]. Our research reveals that exposing 3T3 cell lines to 300 μM CoCl_2_ significantly boosts their viability, exceeding 73%. We measured HIF-1α expression as a successful CoCl_2_-mediated hypoxia stimulation marker to confirm the hypoxic model’s efficacy in these cells.

### 2.3. Clinical Trial Study

#### 2.3.1. Study Design and Subject Recruitment

**Figure 1:**
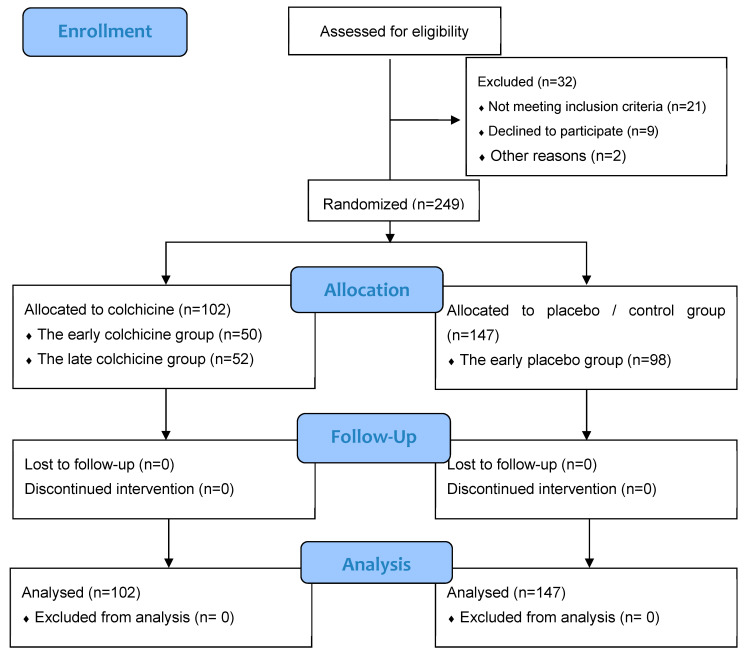
CONSORT 2010 Flow Diagram for our clinical trial study [28].

The investigative framework of this study is anchored in an observational design, integrated within clinical trials targeting patients afflicted with AMI (Figure 1). These patients were subject to primary percutaneous coronary intervention (PCI) executed within 12 h of symptom onset. This expansive, multicenter research was conducted across several esteemed Cardiology Departments: Dr. Saiful Anwar Hospital in Malang City, Dr. Soebandi Hospital in Jember Regency, Binasehat Hospital in Jember Regency, Kaliwates General Hospital in Jember Regency, and Dr. Iskak Hospital in Tulungagung Regency. Data collection spanned from September 2022 to February 2023. In this randomized, double-masked trial, participants were allocated to either a control cohort receiving optimal medical therapy with a placebo or an intervention cohort treated with the optimal medical therapy complemented by colchicine administration. The sample pool included 249 patients, stratified as follows: 98 in the early placebo group, 50 in the early colchicine group, 49 in the late placebo group, and 52 in the late colchicine group. Sampling assessments were conducted at 24 h and 5 days post-intervention. Ethical clearance for this study was granted under the local ethical approval number 400/235/K.3/302/2020, conferred by Saiful Anwar Hospital, Malang, Indonesia. Further to its ethical compliance, this study was registered with the international study registry ISRCTN, bearing the registration number ISRCTN12958502.

#### 2.3.2. Enzyme-Linked Immunosorbent Assay (ELISA) Assay

Sera from Peripheral Blood Mononuclear Cell culturing was subjected to ELISA using the Human Interleukin 10, IL-10 ELISA Kit (BT Lab, Bioassay Technology Lab, Shanghai, China, Cat No: E0102Hu) [29].

#### 2.3.3. Data Analysis

The HIF-1α and IL-10 data were subjected to One-way ANOVA (*p* < 0.05) followed by post hoc Tukey’s test for comparisons. IL-10 levels in sera were analyzed using a T test to determine the difference between control group and intervention. The statistical analysis was generated using GraphPad Prism version 10.0.2 (Software, Inc., San Diego, CA, USA).

## 3. Results

### 3.1. In Silico Study

#### 3.1.1. Prediction of Colchicine Potential Based on SAR

**Figure 2:**
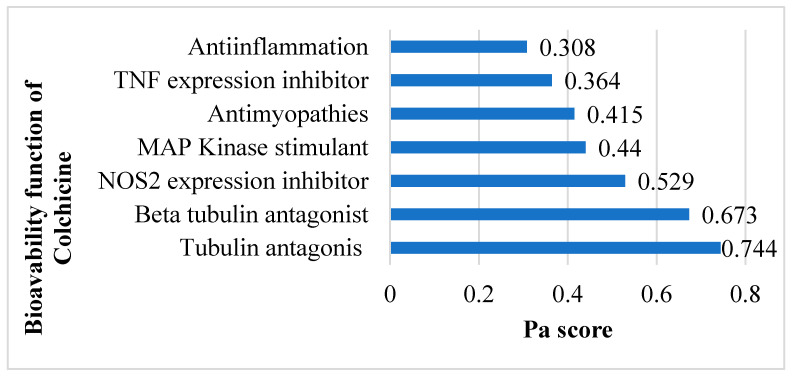
Potential bioavailability of colchicine. Colchicine actively combats cardiac fibrosis and myocardial infarction by harnessing its anti-inflammatory and preventing excessive tubulin formation.

The SMILES profile for colchicine is CC(=O)NC1CCC2=CC(=C(C(=C2C3=CC=C(C(=O)C=C13)OC)OC)OC)OC. A compound is considered to have very high biological activity based on laboratory-scale tests when the Pa (Probability active) value exceeds 0.7 and is greater than Pi (Probability inactive), implying a significant resemblance to drug compounds in terms of similar bioactivity [30]. According to the SAR analysis, colchicine is predicted to act as a tubulin antagonist (0.744), beta-tubulin antagonist (0.673), NOS2 inhibitor (0.529), MAP kinase stimulant (0.44), antimyopathies (0.415), TNF expression inhibitor (0.364), and anti-inflammatory (0.308) (Figure 2) related to cardiac fibrosis and myocardial infarction.

#### 3.1.2. Prediction of Targets and Genes Related to Colchicine, AMI, and CF

**Figure 3:**
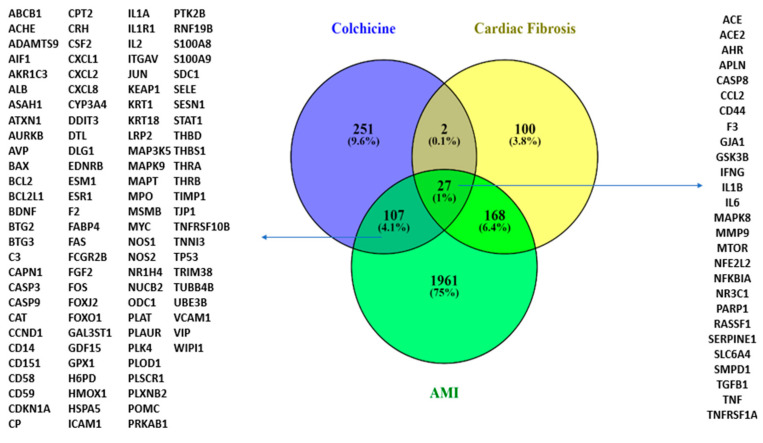
A Venn diagram illustrating colchicine gene targets in CF and AMI.

Following the SAR analysis, we performed a modelling analysis to explore possible colchicine targets against CF and AMI. Exploring colchicine targets using CTD and DIGEP-Pred and Gene Disease Association with DisGeNET and Open Target revealed that colchicine shares 27 targets with CF and AMI. IL-10 interacts with IL-1β, IFN-γ, CCL2, TNF, and TGF-β1 in AMI and CF conditions (Figure 3).

The targets of colchicine include NR3C1 and IL-10. IL-10 interacts with IL-1β, IFN-γ, CCL2, TNF, and TGF-β1 under conditions associated with AMI and cardiac fibrosis.

**Figure 4:**
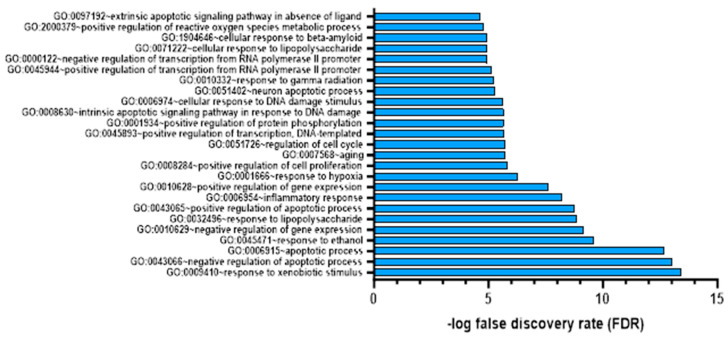
Gene target colchicine annotation with FDR < 0.05.

We annotated each colchicine target using the DAVID web server to identify the functions of these gene targets. As shown in Figure 4, the top 20 targets of colchicine in cardiac fibrosis and AMI represent their impact on biological responses like hypoxia and inflammation. To verify the reliability of our microarray analysis data, we considered genes with a false discovery rate (FDR) of less than 0.05 as significantly different [31]. 

### 3.2. In Vitro Study

#### 3.2.1. HIF-1α Expression

**Figure 5:**
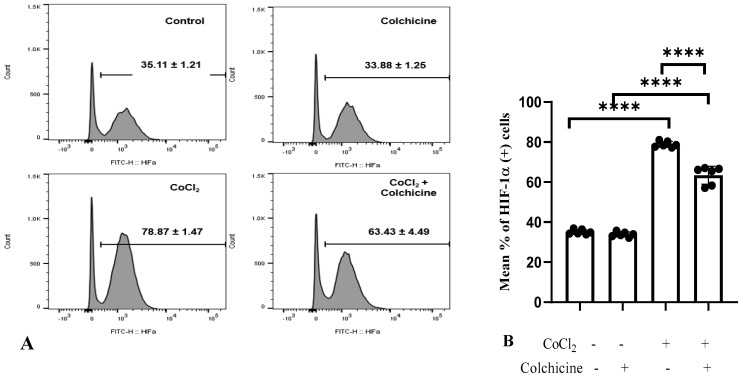
HIF-1α expression in the treatment groups. (**A**,**B**). (1) Control: The cell culture is not exposed to colchicine or CoCl_2_. (2) Administration of 300 µM CoCl_2_ for 24 h significantly increased HIF-1α expression in 3T3 cells compared to the control. (3) Administration of colchicine to cells not experiencing hypoxia (without CoCl_2_) resulted in a reduction in HIF-1α compared to the control, but it was not statistically significant. (4) Administering colchicine for 3 h post-hypoxia (CoCl_2_ 24 h) reduced HIF-1α expression compared to that in the CoCl_2_ (hypoxia) group. **** *p* < 0.00.

Our data showed that CoCl_2_ treatment significantly increased the percentage of HIF-1α-positive cells (78.87 ± 1.47%) compared to the control/normoxia group (35.11 ± 1.21%, *p* < 0.0001, Figure 5A).

Furthermore, colchicine treatment did not affect HIF-1α expression in normoxic-3T3 cells (*p* > 0.05). On the other hand, CoCl_2_ treatment significantly suppressed HIF-1α expression in hypoxic-3T3 cells (63.43 ± 4.49% vs. 78.87 ± 1.47%, *p* < 0.0001, Figure 5B), suggesting that colchicine has a protective role in alleviating the hypoxia state in 3T3 cells.

#### 3.2.2. IL-10 Expression

**Figure 6:**
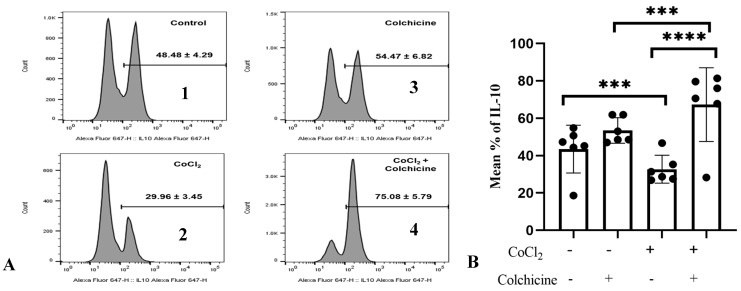
A. IL-10 expression in the treatment groups. (**A**) (1) Control: The cell culture is not exposed to colchicine or CoCl_2_. (2) Administration of 300 µM CoCl_2_ for 24 h significantly reduced IL-10 expression in 3T3 cells compared to the control. (3) Administration of colchicine to cells not experiencing hypoxia (without CoCl_2_) resulted in an increase in IL-10 compared to the control, but it was not statistically significant. (4) Administering colchicine for 3 h post-hypoxia (CoCl_2_ 24 h) increased IL-10 expression compared to that in the CoCl_2_ (hypoxia) group. (**B**). IL-10 expression was assessed using a one-way ANOVA test. The control group did not significantly differ from the colchicine group but showed significant differences when compared to both the CoCl_2_ group and the CoCl_2_ group. *** *p* < 0.001, **** *p* < 0.0001.

To confirm the association between colchicine and the IL-10 pathway in fibroblasts, we investigated IL-10 expression in 3T3 cells exposed to hypoxia and colchicine. Cells were first treated with CoCl_2_ (300 µM) for 24 h to induce hypoxia, mimicking the conditions studied in previous experiments. Colchicine (10 µM) was added, and the IL-10 levels were measured 24 h later using flow cytometry (Figure 6A).

Our data showed that hypoxia stimulation significantly reduced the percentage of IL-10-positive cells compared to normoxic cells (29.96 ± 3.45% vs. 48.48 ± 4.29%, *p* < 0.0001, Figure 6), suggesting that hypoxia stimulation suppressed the anti-inflammatory properties of 3T3 cells. Interestingly, IL-10 expression in colchicine-treated hypoxic cells was significantly enhanced compared to its corresponding control group (75.08 ± 5.79% vs. 48.48 ± 4.29%, *p* < 0.0001, Figure 6B), indicating that colchicine treatment inhibits proinflammatory properties of fibroblast cells.

### 3.3. Clinical Trial Study

#### Baseline Characteristics of AMI Patients

The baseline patient profile in this heart disease study reflects a diverse demographic and clinical landscape as it can be seen in the Table 1. The average age was in the late fifties, showing slight intergroup variation, with no statistically significant differences in age (*p* > 0.05). The majority of participants were male, with more than three-quarters in each group, and there was no significant variance in the gender distribution (*p* > 0.05). In terms of risk factors, a substantial proportion were former smokers, with the highest percentage in the early placebo group (87.76%) and the lowest in the late colchicine group (50%), though without statistical significance (*p* > 0.05). The prevalence of hypertension was consistent across all groups, with no meaningful differences (*p* > 0.05). The incidences of Diabetes Mellitus (DM) and dyslipidemia were relatively low, with the highest occurrence of DM in the late colchicine group (23.08%) and the lowest in the early and late placebo groups at around 10%. Dyslipidemia levels ranged from 9.62% to 22%, again with no significant differences (*p* > 0.05).

Regarding the infarct-related artery, involvement of the left anterior descending (LAD) artery varies between 29.59% and 51.92%, and non-LAD arteries between 40.82% and 70.41%, with no significant differences between groups (*p* > 0.05).

The table presents the demographic and clinical characteristics of cardiac patients enrolled in a study assessing the effects of colchicine. The data indicate an average age in the late fifties, a predominance of male gender, and a uniform distribution of cardiovascular risk factors—such as smoking history, hypertension, diabetes, and dyslipidaemia—across both the intervention and control groups with no significant differences (*p* > 0.05). Furthermore, there was no statistically significant variation in the involvement of infarct-related arteries (both LAD and non-LAD) across all groups (*p* > 0.05), establishing a consistent baseline for subsequent response evaluations to colchicine treatment.

**Figure 7:**
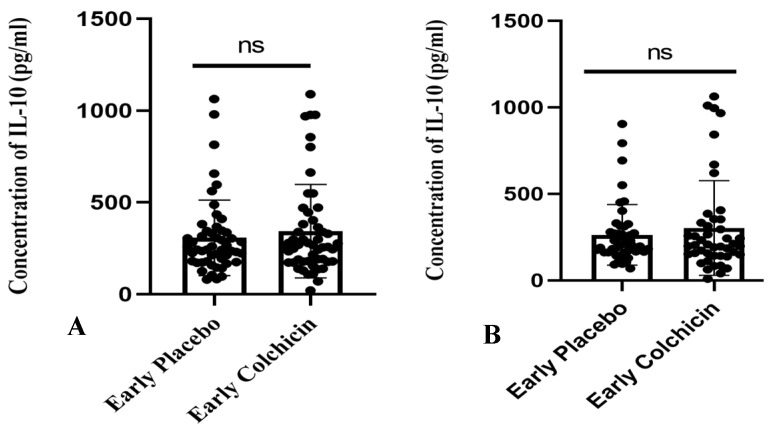
Figure illustrates the administration of both placebo and colchicine at early 24 h (**A**) and five days (**B**) in AMI patients. It demonstrated no significant difference in IL-10 levels (*p* > 0.05). ns: no significance. IL-10 expression was assessed using a *T*-test.

The research findings suggested that administering colchicine to early AMI patients for both 24 h and five days did not result in a significant difference in IL-10 levels between the treatment groups (*p* > 0.05, Figure 7). However, it is noteworthy that the colchicine-treated group exhibited a tendency towards increased IL-10 levels compared to the non-colchicine group. This trend was observed at both the 24 h (343.3 ± 35.28 vs. 307.7 ± 28.78) and five-day time points (302.2 ± 40.81 vs. 263.2 ± 25.82).

**Figure 8:**
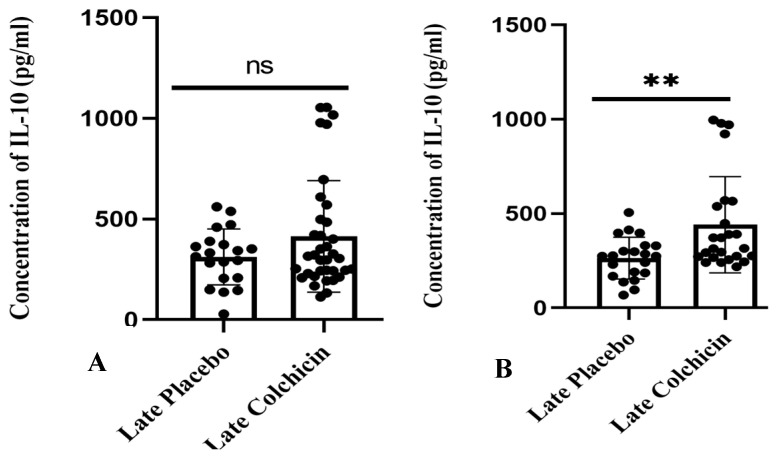
Depicts late colchicine administration at 24 h (**A**) showed no significant difference (*p* > 0.05), while at five days (**B**), there was a significant difference in IL-10 levels. ns: no significance. IL-10 expression was assessed using a *T* test.

In contrast, a separate study found that administering colchicine to AMI patients 24 h late did not result in a significant difference in IL-10 levels between the treatment and control groups (*p* > 0.05, as shown in Figure 8A). However, the mean IL-10 level in the colchicine group displayed a trend towards being higher than the control group (413.8 ± 46.17 vs. 311.5 ± 31.02), as illustrated in Figure 8. Nevertheless, the mean IL-10 level after the administration of colchicine five days late significantly differed, showing higher IL-10 levels compared to the non-colchicine group (*p* < 0.01, as shown in Figure 8B), with average levels of 440.7 ± 50.95 vs. 263.8 ± 24.25).

## 4. Discussion

Managing inflammatory risk factors is crucial for preventing disease progression and improving patient outcomes [32,33,34,35,36,37,38]. Colchicine is predicted to play a significant role in cardiac fibrosis and myocardial infarction, with a score greater than 0.5 as a tubulin antagonist, beta-tubulin antagonist, and NOS2 inhibitor. Colchicine’s mechanism of action involves binding to tubulin, thus hindering microtubule assembly and polymerization. This prediction aligns with the research conducted by McLoughlin and O’Boyle, which states that colchicine works by blocking β-tubulin and subsequently disrupting microtubule assembly [39]. Microtubules, crucial components of the cytoskeleton, are involved in various cellular processes, including maintaining cell shape, intracellular transport, cytokine and chemokine secretion, cell migration, ion channel regulation, and cell division, and are responsible for the subcellular arrangement of mitochondria and cardiac muscle cell dynamics [40]. Colchicine inhibits microtubule growth at low concentrations, while higher concentrations induce microtubule depolymerization. Microtubule depolymerization disrupts neutrophil adhesion and recruitment to inflamed tissues [41].

Colchicine targets related to CF and AMI impact biological responses such as hypoxia and inflammation. Hypoxia or ischemia disrupts endothelial cell barrier integrity, increasing blood vessel permeability and initiating leukocyte infiltration. If ischemia persists for an extended period, it can activate programmed cell death and myocardial cell necrosis, including necrosis, apoptosis, and autophagy mechanisms. Subsequent reperfusion exacerbates tissue damage due to sudden reoxygenation of reactive oxygen species (ROS) generation and complement pathway activation [39]. Necrotic cells and stressed/damaged extracellular matrices release danger-associated molecular patterns (DAMPs) and activate a series of inflammatory mediators, including inflammatory cytokines, chemokines (such as monocyte chemoattractant protein-1/chemokine (C-C motives) ligand 2 [CCL2]), and cell adhesion molecules. In the case of MI, this signalling will activate mitogen-activated protein kinases (MAPKs) and nuclear factor (NF)-κB, leading to inflammation [42].

The colchicine target IL-10 interacts with IL-1β, IFN-γ, CCL2, TNF-α, and transforming growth factor (TGF-β1) in conditions such as AMI and CF. Cytokines such as interleukin-1 (IL-1) and monocyte chemoattractant protein-1 (MCP-1) increase during MI [6]. Colchicine reduces inflammatory cytokines and TGF-β1 by inhibiting NF-κB signalling and activating the NLRP3 inflammasome. IL-10 is produced by T cells and macrophages during acute and chronic inflammation, playing a role in inhibiting apoptosis and TNF-α-induced oxidative stress. Receptor-mediated IL-10 signalling in the heart controls myocardial hypertrophy in response to excessive pressure stimulation. IL-10 can also reduce proinflammatory cytokines such as TNF-α, IL-1β, and IL-6, as well as cardiac contractility in heart failure [43].

Hypoxia plays a role in both physiological and pathophysiological conditions. Physiologically, it is involved in processes such as cell differentiation and embryogenesis [44]. Conversely, in pathophysiological scenarios, hypoxia is associated with conditions such as brain and heart ischemia [28]. Myocardial infarction triggers events that lead to the repair and healing of the left ventricle, resulting in scar tissue formation [45]. This process involves critical inflammatory responses necessary for heart repair. However, it also has implications for postinfarction inflammation and heart failure [46].

Cobalt chloride (CoCl_2_) induces hypoxia by reducing oxygen levels [44]. It increases the stability of hypoxia-inducible factor (HIF) 1α, which is linked to changes in cellular oxygen levels [39]. Administration of CoCl_2_ led to a significant decrease in IL-10 expression in 3T3 cells compared to the control group [47]. This research aligns with the findings of Tao et al. showed that IL-10 expression decreases at 4, 8, and 16 weeks after myocardial infarction, indicating a correlation between reduced IL-10 and impaired heart function [19].

The administration of colchicine to nonhypoxic cells did not significantly differ from IL-10 expression in the control group. In the control group, IL-10 expression has functions in tissue repair and energy metabolism [45]. IL-10 suppresses the infiltration of inflammatory cells and the expression of proinflammatory cytokines in the myocardium by inhibiting fibrosis through the suppression of p38 mitogen-activated protein kinase (MAPK) and by increasing capillary density through the activation of signal transducer and activator of transcription (STAT3), which promotes myocardial capillary growth and protects the heart from ischemic injury [5]. The administration of colchicine three hours after hypoxia (after 24 h of CoCl_2_ exposure) also shows an increase in IL-10 expression compared to the hypoxia group. After AMI, leukocytes infiltrate the infarcted area to clear necrotic remnants and respond to myocardial injury by producing proinflammatory cytokines that peak within one-hour post-MI. Between days 3 and 5 post-MI, there was a transition from inflammatory responses to reparative and anti-inflammatory processes, marked by the acceleration of fibroblast activation and proliferation. Prolonged inflammatory responses can disrupt the physiology of the left ventricle (LV), leading to LV dilation and excessive scar tissue formation. Therefore, expediting inflammatory response resolution is critical, and anti-inflammatories such as IL-10 are needed to suppress inflammation and activate profibrotic processes. IL-10 plays a crucial role in controlling inflammation by regulating the function of macrophages to adopt M2 (anti-inflammatory) polarization [48] and dendritic cells [49]. This research aligns with the findings of Stumpf et al. [50].

The clinical trial results align with the in vitro findings, indicating that administering late colchicine to AMI patients can elevate IL-10 levels compared to those not receiving it on the fifth day. CD4^+^ T cells and Foxp^3+^ regulatory T cells infiltrate the myocardium within days after MI [51]. IL-10 acts as an anti-inflammatory agent by inhibiting antigen presentation by macrophages, suppressing T-cell proliferation, deactivating dendritic cells, or inducing regulatory T-cell function [52], thereby reducing inflammatory products [53] to prevent rupture and left ventricular dilation [51]. Colchicine, as an anti-inflammatory agent in AMI conditions, aligns with the research conducted by Kaur et al. (2006), demonstrating that the administration of anti-inflammatory drugs such as steroids and dexamethasone for cardiac function improvement can increase IL-10 levels and decrease inflammatory cytokines like TNF-α and the NF-ĸβ transcription factor [54]. IL-10 post-MI is an anti-inflammatory cytokine that plays a crucial role in preventing inflammatory and autoimmune pathologies, improves the LV microenvironment to decrease inflammation and facilitate healing [6]. Combined, our results and the literature support the idea that regulation of the inflammatory response by elevating IL-10 may provide cardio-protection to prevent inflammation following MI [6].

In conditions of ischemic heart disorders, macrophages deficient in IL-10 contribute to a shift in macrophage phenotype towards a profibrotic subset, activating fibroblasts. Increased fibroblasts and collagen deposition lead to myocardial relaxation disorders, especially diastolic [55]. Myocardial relaxation disorders occur because inflammatory cytokines in fibroblasts, such as IL-1β, TNF-α, and IL-6, inhibit proliferation, reduce matrix synthesis, and increase MMP activity [56]. IL-10 in macrophages also functions to reduce ROS, resulting in a decrease in fibroblast numbers [55].

### Limitation

Our study has several limitations. The population of women in our study was lower (14.29%) than would be expected given the percentage of women with acute myocardial infarction in the general population. We did not collect level of IL-10 at baseline or during the trial, and we cannot report outcomes according to risk-factor control. We did not routinely measure C-reactive protein levels or other laboratory indicators of inflammation at baseline, and we cannot explore the effects of treatment according to inflammatory state at baseline. However, the effects of treatments in our clinical trials were consistent with the results of our in silico and in vitro studies. The results of our clinical trial showed that among patients with acute myocardial infarction, which all patients were already receiving proven cardiovascular therapies, the level of IL-10 was significantly higher with the administration of colchicine five days late compared to the non-colchicine group.

## 5. Conclusions

Colchicine acts as an anti-inflammatory and antifibrotic agent by increasing IL-10 levels in AMI patients, which is supported by in vitro data on 3T3 cell lines and an in silico analysis based on network pharmacology. Colchicine can be used to boost IL-10 levels with anti-inflammatory protection, specifically in the context of managing inflammation and preventing heart failure.

In summary, we conclude that the IL-10 pathway is colchicine’s one potential target for post-MI inflammation, particularly in the development of cardiac fibrosis. In this study, we showed that a combined approach of network pharmacology along with in vitro experiments to validate the findings is beneficial to unravel the mechanism of colchicine in this specific pathological are important factors in CVD. Inflammation management and prompt treatment are crucial for improving patient outcomes. However, further studies are required to understand how colchicine affects cardiac fibrosis development in response to AMI.

## Figures and Tables

**Table 1:** Baseline clinical and echocardiographic AMI patient characteristics.

	Early Placebo	Early Colchicine	*p* Value	Late Placebo	Late Colchicine	*p* Value
Age (years) (Mean ± SD)	57.34 ± 10.75	58.60 ± 9.13	0.479	56.39 ± 10.85	59.08 ± 9.41	0.457
Sex (Male), *n* (%)	84 (85.71%)	42 (84%)	0.810	41 (83.67%)	40 (76.92%)	0.459
Smoker, *n* (%)	86 (87.76%)	43 (86%)	0.800	33 (67.35%)	26 (50%)	0.106
Hypertension, *n* (%)	34 (39.80%)	19 (38%)	0.720	32 (67.35%)	35 (67.31%)	0.837
Diabetes Melitus, *n* (%)	9 (9.18%)	5 (10%)	>1.000	6 (12.24%)	12 (23.08%)	0.197
Dyslipidaemia, *n* (%)	14 (14.29%)	11 (22%)	0.253	5 (10%)	5 (9.62%)	>1.000
Infarct related artery (LAD), *n* (%)	29 (29.59%)	15 (30%)	>1.000	29 (59.18%)	27 (51.92%)	0.670
Infarct related artery (Non LAD), *n* (%)	69 (70.41%)	35 (70%)	>1.000	20 (40.82%)	25 (48.08%)	0.670

## Data Availability

The authors confirm that the data supporting the findings of this study are available within the article.
